# A European-wide dataset to uncover adaptive traits of Listeria monocytogenes to diverse ecological niches

**DOI:** 10.1038/s41597-022-01278-6

**Published:** 2022-04-28

**Authors:** Benjamin Félix, Yann Sevellec, Federica Palma, Pierre Emmanuel Douarre, Arnaud Felten, Nicolas Radomski, Ludovic Mallet, Yannick Blanchard, Aurélie Leroux, Christophe Soumet, Arnaud Bridier, Pascal Piveteau, Eliette Ascensio, Michel Hébraud, Renáta Karpíšková, Tereza Gelbíčová, Marina Torresi, Francesco Pomilio, Cesare Cammà, Adriano Di Pasquale, Taran Skjerdal, Ariane Pietzka, Werner Ruppitsch, Monica Ricão Canelhas, Bojan Papić, Ana Hurtado, Bart Wullings, Hana Bulawova, Hanna Castro, Miia Lindström, Hannu Korkeala, Žanete Šteingolde, Toomas Kramarenko, Lenka Cabanova, Barbara Szymczak, Manfred Gareis, Verena Oswaldi, Elisabet Marti, Anne-Mette Seyfarth, Jean-Charles Leblanc, Laurent Guillier, Sophie Roussel

**Affiliations:** 1 French Agency for Food, Environmental and Occupational Health & Safety (ANSES), Laboratory for Food Safety, Salmonella and Listeria Unit, Paris-Est University, 14 rue Pierre et Marie Curie, 94701 Maisons-Alfort, France; 2 ANSES, Laboratory for Food Safety, Paris-Est University, 14 rue Pierre et Marie Curie, 94701 Maisons-Alfort, France; 3 ANSES, Ploufragan/Plouzané/Niort Laboratory, Viral Genetics and Bio-Security Unit, Université Européenne de Bretagne, Ploufragan, France; 4 ANSES, Antibiotics, Biocides, Residues and Resistance Unit, 10 B rue Claude Bourgelat – Javené, CS 40608, 35306 Fougères, France; 5 INRAE, Unité de recherche OPAALE, 35000 Rennes, France; 6 INRAE, Agroecologie, AgroSup Dijon, INRA, Univ. Bourgogne Franche-Comté, 21000 Dijon, France; 7 INRAE, UCA, UMR MEDiS, F-63122 Saint-Genès Champanelle, France; 8 Veterinary Research Institute, Hudcova 70, 621 00 Brno, Czech Republic; 9 IZSAM, Istituto Zooprofilattico Sperimentale dell’Abruzzo e del Molise G.CaporaleVia Campo Boario, 64100 Teramo, Italy; 10 Norwegian Veterinary Institute, Food Safety and Animal Health Research, Ullevålsvegen 68, 0454 Oslo, Norway; 11 AGES Austrian Agency for Health and Food Safety, Spargelfeldstrasse 191, 1220 Vienna, Austria; 12 Department of Biology, Swedish Food Agency, Uppsala, Sweden; 13 Institute of Microbiology and Parasitology, Veterinary Faculty, University of Ljubljana, Gerbičeva 60, 1000 Ljubljana, Slovenia; 14 Animal Health Department, NEIKER – Basque Institute for Agricultural Research and Development, Basque Research and Technology Alliance (BRTA), Bizkaia Science and Technology Park 812 L, 48160 Derio, Spain; 15 Wageningen Food Safety Research, Wageningen University & Research, Wageningen, the Netherlands; 16 State Veterinary Institute, Rantířovská 93/20, 586 05 Jihlava, Czech Republic; 17 Department of Food Hygiene and Environmental Health, Faculty of Veterinary Medicine, University of Helsinki, Agnes Sjöbergin katu 2, 00790 Helsinki, Finland; 18 Institute of Food Safety, Animal Health and Environment BIOR, Riga, Latvia; 19 Veterinary and Food Laboratory, F. R. Kreutzwaldi 30, 51006 Tartu, Estonia; 20 State veterinary and food institute DVM, Department of Food Hygiene, Veterinary and food institute, Janoskova 1611/58, Dolny Kubin, Slovakia; 21 Department of Applied Microbiology and Human Nutrition Physiology, West Pomeranian University of Technology, Szczecin, Poland; 22 Chair of Food Safety, Faculty of Veterinary Medicine, Ludwig-Maximilians-University Munich, Oberschleissheim, Germany; 23 Institute of Food Safety and Food Hygiene, Working Group Meat Hygiene, Faculty of Veterinary Medicine, Freie Universität Berlin, Berlin, Germany; 24 Agroscope, Food Microbial Systems, Berne-Liebefeld, Bern, Switzerland; 25 DTU, Technical University of Denmark, National Food Institute, Lyngby, Denmark

**Keywords:** Bacterial genetics, Bacteriology

## Abstract

*Listeria monocytogenes* (*Lm*) is a ubiquitous bacterium that causes listeriosis, a serious foodborne illness. In the nature-to-human transmission route, *Lm* can prosper in various ecological niches. Soil and decaying organic matter are its primary reservoirs. Certain clonal complexes (CCs) are over-represented in food production and represent a challenge to food safety. To gain new understanding of *Lm* adaptation mechanisms in food, the genetic background of strains found in animals and environment should be investigated in comparison to that of food strains. Twenty-one partners, including food, environment, veterinary and public health laboratories, constructed a dataset of 1484 genomes originating from *Lm* strains collected in 19 European countries. This dataset encompasses a large number of CCs occurring worldwide, covers many diverse habitats and is balanced between ecological compartments and geographic regions. The dataset presented here will contribute to improve our understanding of *Lm* ecology and should aid in the surveillance of *Lm*. This dataset provides a basis for the discovery of the genetic traits underlying *Lm* adaptation to different ecological niches.

## Abstract (summary)

**Table:** 

Measurement(s)	whole genome sequencing
Technology Type(s)	Illumina Sequencing
Factor Type(s)	Multi-locus sequence types • Geographic location • Animal associated environment isolates • Food product and food production environment isolates
Sample Characteristic - Organism	Listeria monocytogenes
Sample Characteristic - Environment	Farm • Ruminant • Agricultural soil • Wild animals • food processing building • dairy food product • meat or meat product (from mammal) (us cfr) • chicken meat food product • fish food product • vegetable or vegetable product (us cfr)
Sample Characteristic - Location	Europe

## Background & Summary

*Listeria monocytogenes* (*Lm*) is a facultative intracellular pathogen responsible for listeriosis, a serious disease affecting both humans and animals. *Lm* is a ubiquitous bacterium that is found in various ecological niches, including the natural and farm environments^1,2^. In particular, soil is a primary ecological niche of *Lm* and may thus be important in its transmission from natural/farm environment to food and food-processing environment (FPE)^1,2^. Farm animals, in particular ruminants, are also an additional important reservoir for *Lm* and contribute to contamination of the farm environment through fecal shedding^3,4^. In addition, *Lm* can persist for a long time in soil and the farm environment. Increasing amounts of information are also available on the prevalence of *Lm* in wildlife, showing that various animal species (e.g., deer, wild boars, bears, foxes, monkeys, rodents, hedgehogs, snails, slugs and birds) can act as a vehicles for this pathogen^5–11^. These findings point to an ecological role of wildlife as a reservoir of *Lm* and its potential importance in *Lm* infection cycle.

*Lm* is genetically heterogeneous species divided into four phylogenetic lineages, of which lineages I and II are the most frequently encountered. Multilocus sequence typing (MLST) classifies *Lm* into clonal complexes (CCs) and sequence types (STs), which are systematically used to describe its population structure^12–14^. Certain epidemiological clones account for the majority of outbreaks and sporadic cases in humans^15^ and animals^16^, worldwide^13,17^. The CCs that are commonly found in food and FPE, such as the most common CC9 and CC121, but also CC1, CC2, CC4, CC5, CC6, CC8 and CC37^18^, pose a serious challenge in food industry^15,18,19^. Moreover, they can persist in FPE for several years^20–24^. Remarkably, CC9 and CC121 are rarely reported in animals or natural/farm environments^18,25^.

In order to improve surveillance and the management of health risks associated with *Lm*, a deeper understanding of the genetic make-up of strains adapted to food and FPE is required. As part of the Horizon 2020 “One Health” European Joint Programme, the 3-year research project “LISTADAPT” (Adaptive traits of *Listeria monocytogenes* to its diverse ecological niches - https://onehealthejp.eu/jrp-listadapt/) aimed to identify the genetic mutations and mobile genetic elements underlying the adaptation of *Lm* to different ecological niches. With this objective in mind, strains were collected from i) farm environment and animals and ii) natural environment and wild animals to study their genetic make-up and to compare this background with that of strains isolated from food products and FPE. This work was made possible due to the LISTADAPT consortium which included (i) seven national reference laboratories (NRLs) for surveillance of *Lm* in food, animals and the environment (AT, CZ, DK, FR, IT, NO and SE) and (ii) three research laboratories at INRAE (the French National Research Institute for Agriculture, Food and Environment). Out of the seven NRLs, two are also national public health laboratories (AT and CZ) that are in charge of the surveillance of clinical strains isolated in outbreaks and sporadic cases. In addition, 14 institutes from 12 countries participated as external partners providing isolates.

In this data descriptor, we present a dataset of 1484 high-quality draft genomes originating from *Lm* strains isolated in 19 European countries within the framework of the LISTADAPT project. The constructed dataset cover a wide genetic diversity of *Lm* since it includes about 79 different CCs and singleton STs including the most prevalent CCs in Europe^15^ and worldwide^13,17^. The strains were collected from natural environment (wild animals and natural environment), primary production (farm environment and farm animals with or without listeriosis symptoms) until FPE and food products.

The constructed dataset provides a better understanding of the *Lm* transmission routes from the farm/natural environment to food and FPE and improves our understanding of its ecology. The dataset may also help to assess the importance of animal and food strains for human infection. Moreover, it can be used by the scientific community (i) to improve our understanding of the *Lm* population structure and the *Lm* evolutionary history, (ii) to facilitate the detection of the emerging *Lm* clones and (iii) to identify genetic traits related to the adaptation of *Lm* to particular ecological niches (ecophysiology). Such genetic traits could be used in the development of molecular assays for screening of food/FPE, animal and soil reservoirs.

## Methods

### Construction of the LISTADAPT dataset (n = 1484)

**Fig. 1:**
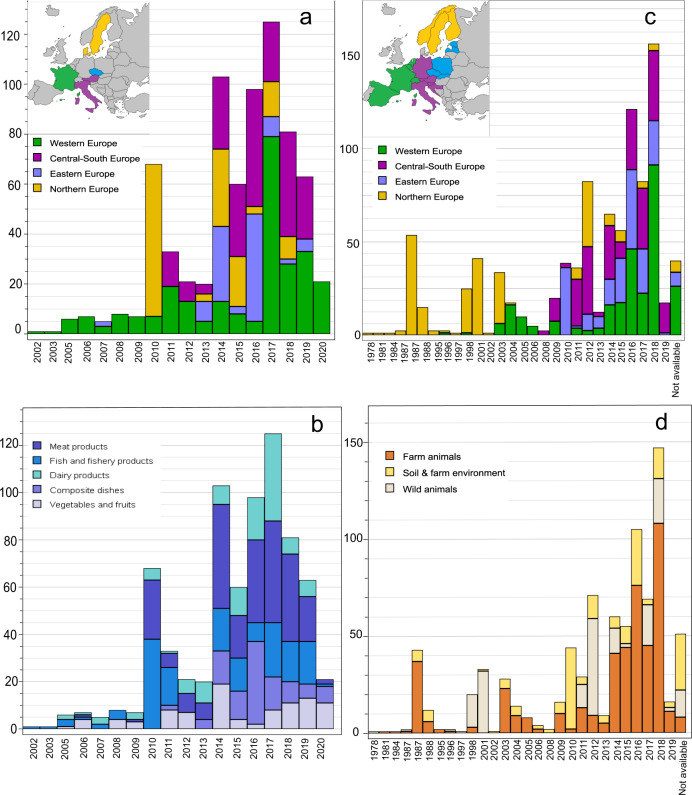
Distribution of the LISTADAPT collection of *Listeria monocytogenes* strains (n = 1484) by time, geographic region and origin of isolation. (**a**) and (**b**) show the distribution of food strains by geographic region and food type, respectively. (**c**) and (**d**) show the distribution of environmental strains by geographic region and subcompartment, respectively.

In order to build a dataset of *Lm* draft genomes suitable for investigating the adaptive traits of *Lm* to diverse ecological niches, we gathered a curated collection of *Lm* draft genomes. Strains isolated over the period 2010–2020 were preferred, regardless of their origin of isolation. We considered two geographic levels, (i) the 27 EU countries including Norway and Switzerland, heterogenous in size, population, climate, ecology and economical activities and (ii) based on country borders four European regions roughly equal in terms of surface area without consideration for other criteria (South-West, Central-South, Eastern and Northern). We included strains that were distributed evenly among these four European regions. The strain were gathered from already available strain collections and extensive sampling campaigns (Fig. 1). The LISTADAPT dataset was divided into two main ecological compartments: (i) C1 compartment, which included strains from animals and the natural/farm environment (n = 756), and (ii) C2 compartment, which included strains from food (n = 728) (Table 1).

**Table 1:** Repartition by compartments and sub-compartments of strains from the whole LISTADAPT collection (1,484).

Animal and environment (C1)				Food products and food production environment (C2)					
Farm animals	Wild animals	Soil and farm environment	Total	Meat	Fish	Dairy	Vegetables and fruits	Composite dish	Total
411	179	166	756	246	165	119	95	103	728

#### Strains selected from the initial collection of the LISTADAPT consortium

At the beginning of the LISTADAPT project, the consortium had access to a collection of about 8000 food and animal *Lm* strains obtained from collaborative projects or national surveillance. Most of these strains were isolated from food, whereas the remainder were isolated from animals (C1 compartment: animal and environmental strains) with a substantial under-representation of certain animal species. This compartment mainly included strains isolated from animals showing listeriosis-related symptoms. Few strains were available from asymptomatic animals, soil and the agricultural environment, originating from three European countries (France, Italy and the Czech Republic).

#### Animal and environmental strains included in the collection during the LISTADAPT project (n = 756)

We collected isolates from animals showing listeriosis associated symptoms, asymptomatic animals, soil and the environment, in a large number of countries across Europe. These strains were isolated between 1978 and 2019. Regarding environmental niches, the consortium selected strains from continental environments remote to cities, large rivers and estuaries or marine environment to avoid the selection of human or food strains released in the environment, detailed strain information were provided in Figshare File 1^26^. However, the six strains described by Szymczak *et al*.^27^ (Table 2) were isolated from city outskirts parks in Poland, distant from the city center. Similarly, the 47 strains from birds (mainly seagulls) (Hellström *et al*.)^10^ were isolated from localities from on the outskirts of Helsinki, Finland (Table 2).

**Table 2:** List of 301 animal and environmental *Listeria monocytogenes* strains from published microbial collections.

Partner	Country (country code)	Category	Origin of isolation	No. of strains	Isolation year	References
Department of Food Hygiene and Environmental Health, Faculty of Veterinary Medicine of Helsinki (n = 131)	Finland (FI)	Wild animals	Wild birds, hare, reindeer [NS]	49	1998, 2001	^10,14^
		Farm animals	Cow, cow milk in bulk tank and pigs [NS]	61	1981–2011	^14,47–50^
			Cow (aborted fetus) [CS]	4	1984–1987	^14,48,49^
		Soil and farm environment	Silage^1^ and soil	17	1987–2004	
Faculty of Veterinary Medicine, University of Munich (n = 31)	Germany (DE)	Wild animals	Deer and wild boar [NS]	31	2011–2012	^6^
Norwegian Veterinary Institute (n = 28)	Norway (NO)	Wild animals	Slugs	28	2012	^11^
Department of Applied Microbiology and Human Nutrition ZUT (n = 36)	Poland (PL)	Soil and farm environment	Soil from agricultural area	30	2010–2012	^27^
			Soil from park on city outskirts	6	2015–2016	
Department of Animal Health, NEIKER (n = 49)	Spain (ES)	Farm animals	Cow, sheep and poultry feces [NS]	49	2004–2005, 2014–2016	^4,51,52^
Veterinary Faculty, University of Ljubljana (n = 26)	Slovenia (SI)	Farm animals	Cow and sheep [CS]	19	2011–2015	^25^
		Soil and farm environment	Farm environment, water, pond	2	2008, 2014	
		Wild animals	Fox brain, deer [NS]	5	2014	

CS, Clinical Symptoms. The reported clinical symptoms included rhombencephalitis, abortion, septicemia and mastitis/subclinical mastitis. The type of clinical samples included cerebellum/brain tissue, aborted fetus, fetal membrane, liver, internal organs, feces and milk.

NS, No listeriosis-associated Symptoms

^1^Strains isolated from silage were considered as originating from the farm environment since silage mainly includes fermented forage crops collected directly from fields.

##### Strains obtained from existing microbial collections (n = 648)

To increase the size and representativeness of the *Lm* genome dataset the LISTADAPT consortium performed an extensive review of all recent collections of published and unpublished *Lm* strains and then contacted researchers in charge of these collections. Finally, 14 external partners, food and veterinary laboratories and research institutes, all dealing with *Lm* hazards in Europe, collaborated with the LISTADAPT consortium (Tables 2 and 3).

**Table 3:** List of 347 animal and environment *Listeria monocytogenes* strains from non-published collections.

Partner	Country	Category	Origin of isolation	No. of strains	Isolation year
Not communicated by the authors (n = **87**)	Belgium (BE)	Farm animals	Cow [NS]	87	2017–2018
Veterinary Research Institute (n = **14)**	Czech Republic (CZ)	Farm animals	Cow, pig [NS]	6	2013–2017
		Soil and farm environment	Mud, algae from pond, soil from farm, decaying vegetation	8	2010, 2014
State veterinary institute (n = **7**)	Czech Republic (CZ)	Wild animals	Gerbil, mouflon [NS]	3	Unknown
		Farm animals	Cow, sheep [NS]	4	Unknown
Veterinary and Food Laboratory (n = **25**)	Estonia (EE)	Farm animals	Cow, sheep, goat [CS]	24	2014–2018
		Wild animals	Deer [CS]	1	2018
Faculty of Veterinary Medicine/ Department of Food Hygiene and Environmental Health, Helsinki (n = **24**)	Finland (FI)	Farm animals	Cow, pork, goat milk, sheep [NS]	7	1987, 1995, 1998
		Wild animals	Hare, birds feces [NS]	4	1986, 1987
		Soil and farm environment	Silage^1^ and farm environment	13	2003, 2014–2015
Laboratory for Food Safety ANSES (n = **25**)	France (FR)	Farm animals	Cow, poultry [NS], horse [CS]	8	2003, 2014, 2015, 2018
		Wild animals	Hare [NS]	3	1986, 1996, 2015
		Soil and farm environment	Manure, soil	14	2004, 2006, 2009, 2012
Research Unit OPAALE INRAE (n = **15**)	France (FR)	Soil and farm environment	Soil, compost, pasture	15	2011, 2012, 2013
Institute of Food Safety and Food Hygiene, Faculty of Veterinary Medicine, Freie Universität Berlin (n = **15**)	Germany (DE)	Farm animals	Pig and sheep at slaughterhouse retention area or immediately after slaughter [NS]	15	2009, 2018–2019
Institute of Food Safety, Animal Health and Environment BIOR (n = **25**)	Latvia (LV)	Farm animals	Cow, goat, sheep, pig [CS]	25	2013–2018
Veterinary Faculty, University of Ljubljana (n = **51**)	Slovenia (SI) (n = 28)	Farm animals	Cow, sheep, goat [CS]	20	2011–2015, 2018–2019
		Wild animals	Fox [NS]	7	2015
		Soil and farm environment	Cattle farm environment	1	2013
	Croatia (HR) (n = 23)	Farm animals	Cow, sheep, goat [CS]	23	2010, 2016–2017
Department of Biology, Swedish Food Agency (n = **5**)	Sweden (SE)	Wild animals	Deer, rook, moose, wild boar [NS]	4	Unknown
		Farm animals	Poultry [NS], goat, sheep [CS]	1	Unknown
State Veterinary and Food Institute Dolny Kubin (n = **22**)	Slovakia (SK)	Farm animals	Sheep, goat [CS]	20	2016–2018
		Soil and farm environment	Feed	2	2015, 2017
Laboratory Feed and Food and Product Safety VWA (n = **32**)	The Netherlands (NL)	Farm animals	Cow, sheep, goat, poultry [NS]	32	2016–2018

CS, Clinical Symptoms. The reported clinical symptoms included rhombencephalitis, abortion, septicemia and mastitis/subclinical mastitis. The type of clinical samples included cerebellum/brain tissue, aborted fetus, fetal membrane, liver, internal organs, feces and milk.

NS, No listeriosis-associated Symptoms

^1^ Strains isolated from silage were considered as originating from the farm environment since silage mainly includes fermented forage crops collected directly from fields.

The initial collection included more strains from animals with listeriosis-associated clinical symptoms than without symptoms. In order to reduce the number of strains originating from animals with listeriosis while maintaining maximum diversity of the dataset, we adopted an original method to select the strains based on metadata (e.g., type of sample, geographic location, time of isolation, molecular typing data such as PFGE profiles, animal species and geographic sampling location). This method relies on Gower’s coefficient (GC), which is a dissimilarity measure: the “distance” between two units is the sum of all the variable-specific distances (associated with metadata categories). The GC metric enables the combination of numeric and categorical data and enables applying weights to each variable, effectively altering the importance of each metadata category (e.g., geographical region as a more important category than year of isolation). The three steps are: (i) calculating the dissimilarity matrix based on Gower’s distance (ii) clustering the dissimilarity matrix with hierarchical clustering (agglomerative bottom-up approach of clustering) and (iii) assessing clusters with the “Silhouette” method. The silhouette plot displays a measure of how close each point in one cluster is to the points in the neighboring clusters. An R script available at https://github.com/lguillier/LISTADAPT/tree/master/metadata2assocation was used to perform the selection of strains based on this method. This script takes as input a Comma Separated Values (CSV) file that includes strain ID and metadata information, then outputs a CSV file of selected strains.

In the present study, we constructed a large dataset comprising 301 animal and environmental *Lm* strains from six European countries and published collections (Table 2), as well as 347 animal and environmental *Lm* strains from 12 European countries that were obtained from non-published collections (Table 3).

##### Strains collected from sampling campaigns (n = 108)

**Fig. 2:**
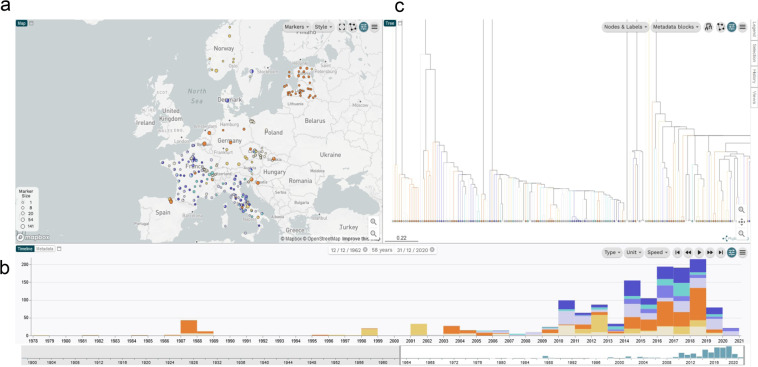
Microreact screenshot representing the distribution of the whole LISTADAPT dataset (n = 1484) by geographic region (**a**) and time (**b**). The k-mer-based phylogenomic clustering of the complete dataset is shown in (**c**). Interactive access to strain metadata and MLST types is available through Microreact^44^, a recently developed online tool for visualizing and sharing spacio-temporal and genetic distributions of strains (Fig. 2, accession link: https://microreact.org/project/8YtGBqEqhosJtysXTVY79M-figure-2-distribution-of-the-whole-listadapt-dataset-n1484-by-geographic-region-time-and-genetic-diversity). The dataset interactive map was generated using either the exact GPS coordinate, regional GPS coordinate or national GPS coordinate according to the level of details available for each strain. An annual timescale was used. The core genome MLST (Moura *et al*.) tree was generated from the draft genome assemblies using pairwise categorical difference and single linkage method in BioNumerics. The tree revealed three main clades corresponding to *Lm* phylogenetic lineages. Each clade included several clusters corresponding to MLST types (CC and singleton ST). Circles in shade of blue show food product isolates (clear blue: fish product, greeblue: dairy products, blue: composite dishes, deep blue: meat products). Circles in shade of orange show animal and environment isolates (beige: soil & farm environment, golden: wild animal, deep orange: farm animals). Circles size is proportional to the number of strains included.

Soil, farm, and wild animal samples were collected in nine European countries (Table 4). For the collection of soil samples, the LISTADAPT project members raised awareness and organised crowd-sampling campaigns. All the soil samples were collected from agricultural or wild areas according to a common procedure provided to the samplers based on the existing recommendations reported in the literature^2,28–30^. The integration of feedback from samplers enabled a continuous improvement of the sampling protocol. The sampling campaigns were conducted in 17 areas in seven EU member states, Norway and Switzerland (Figs. 1 and 2, Table 4), namely AT, CH, CZ, FR, IT, NO, SE, SI and SK, resulting in the isolation of 58 *Lm* strains. Out of the 1752 available sampling records, the overall prevalence was 3%. We confirm in the present study the low prevalence of *Lm* in soil reported in the literature (below 1% and up to 6% depending on soil type)^2,29^. Soil strains from AT, FR, SI and SE were isolated by employing a two-step specific enrichment: the first enrichment was performed with modified *Listeria* Enrichment Broth for 24 h at 30 °C, followed by enrichment in University of Vermont Medium (UVM) enrichment broth for 48 h at 30 °C. Detection of *Listeria* spp. and *Lm* was then achieved by specific SYBR Green real-time PCR targeting *prs2* and *inlA* genes, respectively. The samples positive for the presence of *Listeria* spp. and/or *Lm* were spread on RAPID’L.Mono agar plates (BioRAD, France). After 24 h incubation at 37 °C, colonies characteristic of *Lm* and other *Listeria* species were picked, purified and stored at –80 °C in Tryptone Soya Broth supplemented with 25% (v/v) glycerol. Strains from CH, CZ, IT and SK were isolated with the EN ISO 11290-1:2017 protocol (Horizontal method for the detection and enumeration of *Lm* and of *Listeria spp*.).

**Table 4:** List of 108 animal and environment strains from sampling campaigns.

Partner	Country (country code)	Category	Origin of isolation	No. of strains	Isolation year
Austrian Agency for Health and Food Safety AGES (n = **1**)	Austria (AT)	Soil and farm environment	Meadow	1	2018
Veterinary Research Institute (n = **21**)	Czech Republic (CZ)	Natural and farm environment	Soil, river bank, pond, decaying vegetation, manure	18	2016–2018
	Slovakia (SK)	Natural and farm environment	Soil, river bank	3	2016
Research Unit OPAALE INRAE (n = **13**)	France (FR)	Soil and farm environment	Soil, compost, pasture	13	2018–2019
Istituto Zooprofilattico Sperimentale dell’Abruzzo e del Molise G.Caporale (n = **59**)	Italy (IT)	Farm animals	Cow, goat, sheep [CS]	7	2014–2018
		Soil and farm environment	Soil and river water	16	2016–2018
		Wild animals	Fox, wolf, porcupine, badger, bear, snail, crayfish, roe deer, wild boar [NS]	36	2014, 2016–2018
Norwegian Veterinary Institute (n = **5**)	Norway (NO)	Wild animals	Deer and reindeer [NS]	5	2017–2018
Veterinary Faculty, University of Ljubljana (n = **3**)	Slovenia (SI)	Soil and farm environment	Water pond and soil	3	2018
State Veterinary and Food Institute Dolny Kubin (n = **1**)	Slovakia (SK)	Wild animal	Snail	1	2019
Department of Microbiology, National Food Agency (n = **1**)	Sweden (SE)	Soil and farm environment	Pasture	1	2018
Agroscope (n = **4**)	Switzerland (CH)	Soil and farm environment	Pasture soil	3	2019
		Wild animal	Snail	1	2019

CS, Clinical Symptoms. The reported clinical symptoms included rhombencephalitis, abortion, septicemia and mastitis/subclinical mastitis. The type of clinical samples included cerebellum/brain tissue, aborted fetus, fetal membrane, liver, internal organs, feces and milk.

NS, No listeriosis-associated Symptoms

Regarding the subcompartments of farm and wild animal, 50 *Lm* strains were isolated from sampling campaigns. Three campaigns targeting shelled gastropods sampled in IT, SK and CH resulted in the isolation of six strains (Figs. 1 and 2, Table 4). Sampling campaigns were also carried out for wild deer and reindeer feces in Southern Norway, and from cattle, roe deer, wild boar, wolf, bear and fox feces in the Abruzzo and Molise regions of Italy (Fig. 1, Table 4). Of the 2577 samples collected from vertebrates during the campaign conducted in IT and NO 41 isolates were detected, with an overall prevalence of 1.6%.

#### Food strains included in the collection during the LISTADAPT project (n = 728)

The food strains (C2 compartment) were classified according to the five main categories of risk food matrices for *Lm* defined by the European Food Safety Authority (EFSA)^31^: dairy products (n = 119), fish and fishery products (n = 165), meat products (n = 246), vegetables and fruits (n = 95), and composite dishes (food products combining several food categories) (n = 103). Six NRL project partners (AGES, ANSES, DTU, IZSAM, SLV and VRI) were instructed to target a maximum of 30 strains per food category from their strain collections, preferring strains isolated in the last 10 years. This time period was extended to the under-represented categories (vegetables and fruits); the final dataset included strains originating from the 2002–2020 period. We excluded raw materials from the selection based on the assumption that they could be contaminated by strains originating from farms or animals. The 728 strains from C2 compartment were isolated along the food chain, from food processing plants to food retail in several EU countries (Table 1), detailed strain information were provided in Figshare File 1^26^

### Complete LISTADAPT dataset (n = 1484)

The final LISTADAPT strain dataset that we constructed in collaboration with external partners was balanced with regard to the two main compartments: C1 (animals/environment, n = 756) and C2 (food/FPE, n = 728) (Table 1). The geographic distribution covered 19 of the 27 EU countries plus Norway and Switzerland (Figs. 1 and 2).

Although the C1 compartment (n = 756) covered a 41-year period (1978–2019), most of the strains (75%) were isolated since 2010. This panel covered all successive years between 2009 and 2019 in at least three European regions (Fig. 1c). Between 2008 and 2019, except for the year 2013, the C1 compartment covered all successive years in the following three categories of subcompartments: farm animals, wild animals and natural/farm environment (Fig. 1d).

Although the C2 compartment (n = 728) covered an 18-year period (2002–2020), most of the strains (78%) were recent, i.e. having been isolated between 2013 and 2019 (Fig. 1b). This panel covered all successive years between 2013 and 2019, as well as the five major categories in at least three European regions (Fig. 2b).

Strains from C1 compartment were isolated from more countries (n = 18) than strains from C2 compartment (n = 6). Finally, the majority (1135 of 1484 strains, 76%) of strains from both compartments originated from the period 2011–2019 (Fig. 1a,c).

**Fig. 3:**
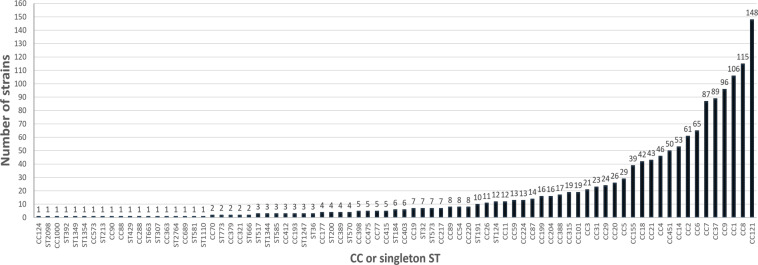
Distribution of the LISTADAPT dataset of *Listeria monocytogenes* genomes by multilocus sequence typing clonal complex (CC) or singleton sequence type (ST).

Overall, the 1484 strains clustered into 137 MLST STs, which belonged to 54 CCs and 25 singleton STs (Fig. 3). For 22 strains, the allele profile was unknown (novel ST) or incomplete (When six out of seven MLST alleles were present, a CC was assigned when possible).

### Standard strain nomenclature

In order to facilitate data sharing between partners, we adopted a standard nomenclature for strain identification (ID). This nomenclature was used as metadata codification to allow for fast identification of the geographic region and detailed isolation source of the strains (e.g., wild animal, food product or farm environment). In more detail, the LISTADAPT code has between 10 and 15 characters; the first two letters (level 1) correspond to the country code (ISO 3166-1-alpha-2 code), which is followed by a code detailing the origin of the strain and the sample type (level 2 to 4, depending on the nature of the source). Briefly, level 2 details the type of sample (e.g., animal species, environment and food categories) and level 3 details the nature of the sample (e.g., type of animal sample, type of food and nature of environmental sample). The level 4 gives additional information about the sample (e.g. type of preparation for aliments and health status of the animals). The code ends with a sequential number for each country, generated when the strain was added to the collection. For example: the strain DE-RDE-CP-13 was isolated in Germany (DE) from a roe deer (RDE) as a clinical strain (CP) and it was the 13^th^ strain isolated from Germany included in the dataset. The Supplementary Table S2 provides a detailed overview of the employed LISTADAPT codification.

### Whole Genome Sequencing and genomes data analysis

The next generation sequencing (NGS) paired-reads (2 × 150 bp) were generated during the project with Illumina platforms. Four LISTADAPT partners (AGES, IZSAM, ANSES and DTU) mainly performed the sequencing. Figshare File 1^26^ lists the sequencing technology and the center which performed the library preparation and produced the sequences.

The genomes were all *de novo* assembled and annotated with a harmonized in-house workflow named ARTwork (Assembly of reads and typing workflow)^32^ used in the ANSES Laboratory for Food Safety. In addition to *de novo* assembly, the ARTwork pipeline also performs genome annotation using Prokka^33^. This whole genome sequencing (WGS) workflow has been described in detail in previous publications^32,34–36^, including the integrated bioinformatics tools and their corresponding versions, enabling repeatability and comparability of the results^2^ (Table 5). Assembled genome files were made publicly available in FASTA format through Figshare^37^.

**Table 5:** Bioinformatics tools used and their versions.

Application		Software	Version
ARTwork			
Read mapping		BBMap	38.22-0
Read normalization		BBNorm	38.22-0
Quality assessment of reads		FastQC	0.11.8
Trimming of low-quality reads		Trimmomatic	0.38
*De novo* assembly		SPAdes	3.13.0
MLST prediction		MLST	2.16.1
Retrieval of the closest reference		Mash	2.0
Reference-based scaffolding		MeDuSa	1.3
Gap closing		GapCloser	2.04
Trimming of contigs < 200 bp		Biopython	
Quality assessment of the assembly		QUAST	5.0.2
Genome annotation		Prokka	1.13.3
**Licensed software**			
Core genome MLST		BioNumerics	V7.3
WGS based molecular serotyping		SeqSphere +	V7.0.4

### Quality control of WGS data

Poor-quality reads or assemblies as well as contaminations can significantly affect gene prediction and cluster analyses^38,39^. Different WGS metrics and quality criteria were thus employed in the ARTwork pipeline to ensure high-quality WGS data. Reads with an estimated depth of coverage < 30 × (as estimated by BBmap^40^) as well as contigs and scaffolds with a length of < 200 bp were excluded (n = 22). Draft genomes with a total length outside the range of 2.7–3.3 Mb and with a total number of scaffolds > 200 (n = 46) were also excluded. In addition, inter- and intra-species contamination of reads was determined using the recently developed ConFindr software (v0.5.1)^41^. Since recently demonstrated, inter-and-intra species contamination of 10 single nucleotide variants (SNVs) assessed by ConFindr in the conserved core genes does not significantly impact cluster analysis^39^. We decided to exclude all genomes presenting SNVs lower than this cut-off (n = 12) as well as various read- or assembly-related errors (n = 34).

The employed WGS metrics and quality criteria of the complete LISTADAPT genome dataset are reported in Figshare File 1^26^. In total, 114 sequenced genomes were of unsatisfactory quality after quality control and were thus excluded from the final dataset. After quality control of NGS and WGS data, the final LISTADAPT dataset included 1484 genomes.

### Metadata and WGS data sharing

All metadata and WGS data collected herein were centralized and processed with standardized criteria for common nomenclature and NGS/WGS quality control before sharing between project partners. Reads normalized to 100 × coverage, draft assemblies (contigs and scaffolds) and annotated genomes (Genome Feature Format, GFF, and Genbank format, GBK) were also centralized at the MongoDB database located at ANSES (Maisons-Alfort Laboratory for Food Safety) providing quickly available, ready-to-use data.

Raw (non-normalized) reads for all the *Lm* strains sequenced in the LISTADAPT collection (n = 1484) were submitted to the NCBI Sequence Read Archive (SRA) for sharing with the LISTADAPT project’s partners. Raw (non-normalized) reads for 67 *Lm* food strains obtained from previous publications^19,42^ were submitted to the NCBI Sequence Read Archive (SRA) database and were linked to their existing accession numbers in Figshare File 1^26^.

## Data Records

All high-quality WGS data from this data descriptor are available for download at SRA/ENA public repository, including the sequences already available at the beginning of this study^43^. Assembly and annotation files are available through Figshare^44^. Complete metadata and quality check parameters are here reported in Figshare File 1^26^.

## Technical Validation

### Redundant strains

The LISTADAPT dataset was analyzed by core-genome MLST (cgMLST) analysis, using BioNumerics (Table 5) according to a fixed cgMLST scheme consisting of 1748 Moura *et al*. loci^45^. All strains with genomes presenting less than < 7 allele differences (AD), isolated in the same year, as well as sharing the same source of isolation and sharing identical geographic location (same region or country) were considered as redundant. When the latter information was not available, the provider was used instead. Although year of isolation was unknown for four strains, they were marked as redundant because of similar cgMLST (<7 AD). Among the 1484 strains, 157 were identified as redundant. These strains were maintained in the dataset and marked accordingly (Figshare File 1^26^)

### Consistency analysis

The present study includes 648 strains from existing collections and 108 strains isolated in the framework of this study. The strains from historical collections were provided from 19 different laboratories. The management of large strain collections may lead to storage issue such as the isolation of two strains in the same tube. Furthermore, the sequencing of the strains involved several handling that may lead to human error.

For 380 of the 648 strains provided by partners, historical typing data were available. We established links between these typing data provided and the sequence obtained. These typing data were either, conventional serotyping data, molecular serotyping or MLST obtained by individual allele sequencing or mapping from PFGE. For conventional serotype the correspondence with the MLST type obtained from WGS was established following correspondence based on Ragon *et al*.^12^. The correspondence with molecular serotyping was established based on Hyden *et al*.^46^ mapping system using the Software SeqSphere (Table 5). For the strains isolated in Belgium (Table 3) the correspondence with PFGE was applied by our partner, based on the methodology described in Félix *et al*.^18^. For the strains isolated in Finland (Tables 2 and 3), the correspondence with PFGE was applied by our partners according to their in-house mapping methodology. The observed discordances were investigated with the partners. The concerned strains were re-sequenced if needed and discarded when unresolved. All results were reported in the Figshare File 1^26^.

## Supplementary information

Supplementary Table S2

## Data Availability

The ARTwork pipeline, described in the WGS quality control section is publicly available at https://github.com/afelten-Anses/ARtWORK. The employed bioinformatics tools and their versions are specified in Table 5.
