# Supplementary material for: A European-wide dataset to uncover adaptive traits of Listeria monocytogenes to diverse ecological niches
Source: Sci Data. 2022 Apr 28;9:190. doi: 10.1038/s41597-022-01278-6 (PMC9050667; doi:10.1038/s41597-022-01278-6)
Supplement: Supplementary file 1 — Supplementary Table S2 [file 41597_2022_1278_MOESM1_ESM.pdf]

Supplementary table S2: Codification used to generate LISTADAPT strain single identifiers

Country (level 1)	
Austria	AT
Belgium	BE
Bulgaria	BG
Cyprus	CY
Czech republic	CZ
Germany	DE
Denmark	DN
Estonia	EE
Greece	EL
Spain	ES
Finland	FI
France	FR
Croatia	HR
Hungary	HU
Ireland	IE
Italy	IT
Lithuania	LT
Latvia	LV
The Netherlands	NL
Norway	NO
Poland	PL
Portugal	PT
Sweden	SE
Slovenia	SL
Slovakia	SK
Switzerland	CH
United Kingdom	UK
United States	US

animal samples

Animal (level 2)		sample type (level3)		statut (level 4)	
bird	BIR	clinical sample	CP	Healthy	H
cattle	BOV	fecal content	FE	ill	I
Deer	DEE	milk	MI	unknown	U
Fallow deer	FDE	other	OT		
Fox	FOX	skin	SK		
Goat	GOA	tonsil	TO		
Poultry	AVI	unknown	UN		
red deer	RDE				
sheep	OVI				
swine	PIG				
wild boar	WBO				
other	OTH				
Human	HU				

farm environment

farm environment (level 2)		sample type (level3)		associated animal (level 4)	
animal feed	AF	Animal stall	AS	avian	A
farm	FAR	Cultivated field	CF	bovine	B
		feed	FE	goat	G
		grassland/eadow	GL	ovin	O
		Water through, well used for anim	WT	pig	P
		unknown	UN	mixed	M

natural environment

environment (level 2)		sample type (level3)	
naturel environement	NAT	soil	SO
urban	URB	vegetation	VE
farm	FAR	water	WT
		garden plot	GA
		park	PA
		unknown	UN

Food sample

Level 2 : Food type		Level 3 : Associated source		Level 4 : Sample Type	
Fish	FI	Bovine	B	Smoked	SM
Meat	ME	Pig	P	Fresh Meat	FM
Vegetable	VE	Avian/poultry	A	Fresh Fish	FF
Dairy	DA	Ovine	O	Raw Product	RA
Composite dishes	CO	Goat	G	frozen	FZ
		Rabbit	R	Processed Meat	PM
		Fish	F	Deli product	DE
		Shellfish	S	Cheese	CH
		Vegetable	V	Milk	MI
		Fruit	F	Cream	CR
		mixed (more than one type of food)	M	Salad	SA
		unknown	U	Unknown	UN
				Other	OT

food processing environment

processing environment (level 2)		Associated food (level 3)		Sample type (level 4)	
processing environment	PE	Fish	FI	Soil/floor	S
		Meat	ME	Machinery	M
		Vegetable	VE	Drain	D
		Dairy	DA	Unknown	U
		Composite dishes	CO		
